# Deep-learning-based ring artifact correction for tomographic reconstruction

**DOI:** 10.1107/S1600577523000917

**Published:** 2023-03-10

**Authors:** Tianyu Fu, Yan Wang, Kai Zhang, Jin Zhang, Shanfeng Wang, Wanxia Huang, Yaling Wang, Chunxia Yao, Chenpeng Zhou, Qingxi Yuan

**Affiliations:** a Beijing Synchrotron Radiation Facility, X-ray Optics and Technology Laboratory, Institute of High Energy Physics, Chinese Academy of Sciences, Yuquan Road, Shijingshan District, Beijing 010000, People’s Republic of China; b University of Chinese Academy of Sciences, Yuquan Road, Shijingshan District, Beijing 010000, People’s Republic of China; c CAS Key Laboratory for Biomedical Effects of Nanomedicines and Nanosafety and CAS Center for Excellence in Nanoscience, National Center for Nanoscience and Technology of China, Beijing 100190, People’s Republic of China; Tohoku University, Japan

**Keywords:** ring artifact correction, X-ray tomography, deep learning, residual neural network

## Abstract

X-ray tomography has been widely used in various research fields thanks to its capability of observing 3D structures with high resolution non-destructively. However, due to the nonlinearity and inconsistency of detector pixels, ring artifacts usually appear in tomographic reconstruction, which may compromise image quality and cause nonuniform bias. This study proposes a new ring artifact correction method based on the residual neural network (ResNet) for X-ray tomography. The artifact correction network uses complementary information of each wavelet coefficient and a residual mechanism of the residual block to obtain high-precision artifacts through low operation costs. In addition, a regularization term is used to accurately extract stripe artifacts in sinograms, so that the network can better preserve image details while accurately separating artifacts. When applied to simulation and experimental data, the proposed method shows a good suppression of ring artifacts. To solve the problem of insufficient training data, ResNet is trained through the transfer learning strategy, which brings advantages of robustness, versatility and low computing cost.

## Abstract 2

A new ring artifact correction method based on a residual neural network for tomographic reconstruction with superior efficiency and accuracy is presented.

## 1. Introduction

X-ray tomography technology has the advantages of strong penetration ability, high imaging resolution and rich contrast source (Kalender, 2006 ▸; Kareh *et al.*, 2014 ▸; Pfeiffer, 2018 ▸; Sakdinawat & Attwood, 2010 ▸), and is widely used in the fields of medicine, biology, material science and chemistry due to these excellent properties (Fu *et al.*, 2022 ▸; Jiang *et al.*, 2020 ▸; Kareh *et al.*, 2014 ▸; Lee *et al.*, 2021 ▸; Li *et al.*, 2022 ▸). However, because of the nonlinearity and inconsistency of detector pixels, a large number of ring and semi-ring artifacts exist in X-ray tomography, seriously reducing the 3D imaging quality (Paleo & Mirone, 2015 ▸; Croton *et al.*, 2019 ▸; Jha *et al.*, 2014 ▸; Boin & Haibel, 2006 ▸). Therefore, a ring artifact correction method that does not decrease the image resolution is necessary for the reconstruction of reliable high-resolution 3D sample structure. Various methods have been proposed for ring artifact correction. These methods can be divided into two categories. One is based on a specifically designed computed tomography (CT) scanning procedure (Davis & Elliott, 1997 ▸; Hubert *et al.*, 2018 ▸; Pelt & Parkinson, 2018 ▸). For example, continuously changing the relative positions of samples and detectors before each projective acquisition can effectively reduce ring artifacts, but this requires a high-precision positioner and increases acquisition complexity. The other method relies on sinogram stripe removal (Münch *et al.*, 2009 ▸; Vo *et al.*, 2018 ▸; Massimi *et al.*, 2018 ▸; Miqueles *et al.*, 2014 ▸; Titarenko, 2016 ▸; Yan *et al.*, 2016 ▸). Ring artifacts are generated by stripe artifacts in sinogram images. Therefore, if stripe artifacts can also be removed by image post-processing, then ring artifact correction can be achieved. For example, the Fourier–wavelet (FW) correction method, which is one of the most popular correction algorithms, combines wavelet transform and Fourier filtering to remove stripe artifacts in sinogram images. However, this method needs complex parameter adjustment to adapt to different stripe widths, and compromising between resolution and quality is also inevitable. With the recent rapid development of artificial intelligence technology (Bai *et al.*, 2022 ▸), the strip noise removal (SNR) network has also been proposed (Guan *et al.*, 2019 ▸). Due to the neural network’s excellent abilities of feature detection and extraction, this correction method performs ring artifact correction especially well. However, given that the traditional network structure is difficult to achieve a deep network with high accuracy, this method is not quite adapted to strong artifacts.

To overcome the above drawback in existing correction methods, a new ring artifact correction method (RRAC) based on a residual neural network (ResNet) is proposed. This method can use complementary information of wavelet coefficients to remove artifacts while preserving and restoring details of the original image. The main views and contributions of this paper are summarized as follows:

(1) The artifact correction network in this paper is designed based on the residual block, which not only saves operational costs but also improves the accuracy of the network.

(2) The input of the artifact correction network is wavelet coefficients of the sinogram, and its image size is one-quarter of the original image. This input method significantly reduces the operation time and memory consumption. The network output is the artifact in wavelet coefficients. This output mode is not only simpler but also conducive to accurately obtaining the intensity and distribution of artifacts.

(3) To prevent the network from outputting information other than stripe artifacts, this paper also adds a regularization term to the loss function, so that the artifact correction network can more accurately separate the details of the sample from the artifact.

(4) In order to make the RRAC method show high accuracy and strong robustness under limited experimental data, we use the transfer learning strategy to solve the problem of insufficient experimental data.

## 2. Method

### 2.1. Deep-learning-based artifact correction

**Figure 1:**
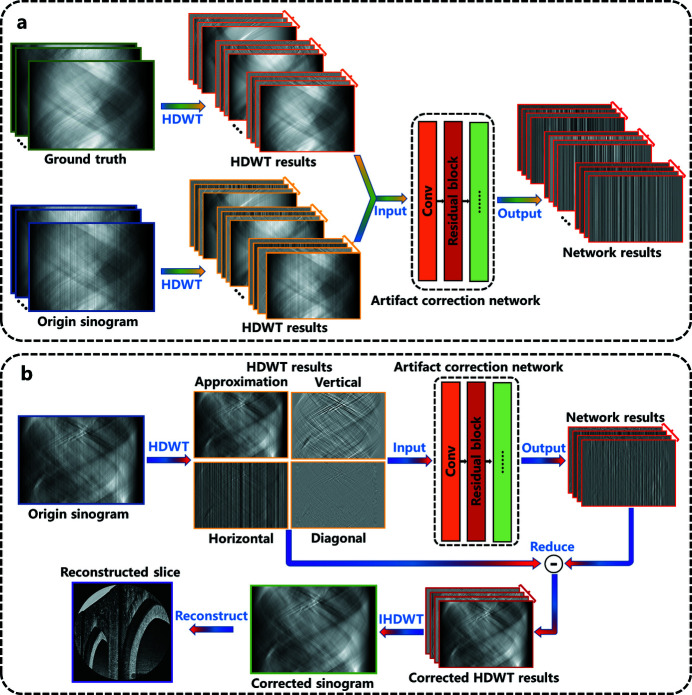
Flow chart of the ring artifact correction method based on ResNet. (*a*) Training process. (*b*) Application process.

The ring artifact correction method based on the ResNet workflow (Fig. 1 ▸) mainly includes two steps: training [Fig. 1 ▸(*a*)] and application [Fig. 1 ▸(*b*)]. The neural network uses a process analogous to the human brain, which requires training a component with labeled data named the training dataset. The training process is as follows: first, the training set data are decomposed into four coefficients by Haar discrete wavelet transform (HDWT) (Lai & Chang, 2006 ▸). Second, the decomposed coefficients are fed into the artifact correction network. The complementary information of different wavelet sub-band coefficients can help the network well preserve and restore the detailed information of an image while eliminating artifacts. Moreover, given that the dimension of the wavelet coefficients is half of the original image, training data pre-processed by HDWT can reduce the required memory space and speed up the training. Finally, the network output is the predicted artifact in a wavelet sub-band coefficient After training, the RRAC method can apply artifact correction to other data without a given ground truth. The application process [Fig. 1 ▸(*b*)] is similar to the training process. First, four wavelet coefficients are generated by the wavelet transform of the original sinogram image. Second, according to these coefficients, the predicted artifact can be produced by the network and, when subtracted from the input wavelet coefficients, clean wavelet coefficients can be obtained. Finally, high-quality reconstruction results without ring artifacts can be generated by inverse Haar discrete wavelet transform (IHDWT) and filtered back projection (FBP) reconstruction (Guersoy *et al.*, 2014 ▸; Pelt *et al.*, 2016 ▸).

### 2.2. Design of the artifact correction neural network

**Figure 2:**
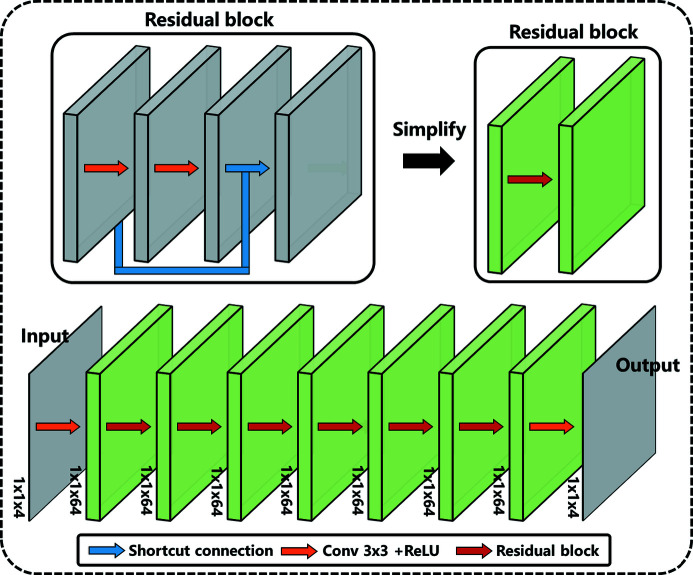
Network structure of the artifact correction network.

The core of the RRAC method is the artifact correction network (Fig. 2 ▸), which is designed on the basis of ResNet (He *et al.*, 2016 ▸). The artifact correction network comprises 14 convolution layers, including two 3 × 3 convolution layers and six residual blocks. Each residual block includes two 3 × 3 convolution layers and one shortcut connection. In the residual block, input data are corrected by the residuals obtained from the two convolution layers. This structural design of the residual block has the following three advantages. First, it can speed up the network training process and save memory space. Second, the residual block can eliminate the problem of vanishing/exploding gradients and consequently be conducive to the establishment of a high-performance deep network (Balduzzi *et al.*, 2017 ▸; Sandler *et al.*, 2018 ▸). Third, the residual is facilitative to image detail preservation. Furthermore, except that the last convolution layer is four channels corresponding to four input wavelet coefficients, the other convolution kernels are 64 channels. Different network layers are connected by the ReLU activation function (Xu *et al.*, 2015 ▸), which brings nonlinear mapping to the network and enables it to deal with nonlinear problems. Moreover, the reason why the size of the image is not changed in the correction network is that the image details will inevitably be lost in the down-sampling process. The purpose of the correction network is to output stripe artifacts in the wavelet coefficients. Most stripe artifacts are single-pixel or several-pixel stripes. The down-sampling will lose some small artifact, which makes it difficult for the artifact correction network to work on some small artifacts.

For the artifact correction network, the loss function can evaluate network output and guide the update of network parameters during the training process, so it plays an important guiding role to the RRAC method. The loss function comprises two terms: wavelet loss function *L*_M_ (Chen *et al.*, 2018 ▸; Huang *et al.*, 2017 ▸) and regular loss function *L*_W_.

The wavelet loss function is the mean square error (MSE) (Ledig *et al.*, 2017 ▸), which is one of the common loss functions and can evaluate well the error between the network output and the ground truth. The MSE is defined as follows,

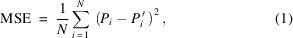

where *N* is the total number of pixels. *P*_*i*_ is the *i*th pixel value of the ground truth, and 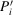 is the *i*th pixel value of the network output. *L*_M_ can be formulated as follows,

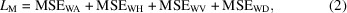

where MSE_WA_, MSE_WH_, MSE_WV_ and MSE_WD_ are the MSE values for the approximation wavelet coefficient (WA), horizontal wavelet coefficient (WH), vertical wavelet coefficient (WV) and diagonal wavelet coefficient (WD), respectively.

The stripe’s gray value varies less along the stripe direction than in the perpendicular direction (Chen *et al.*, 2017 ▸; Liu *et al.*, 2016 ▸). Therefore, the regular loss function *L*_W_ adapts smoothness in the fringe direction to estimate the fringe noise and is defined as follows,

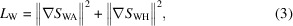

where ∇ denotes the partial differential operator along the stripe direction. *S*_WA_ is the stripe component of WA, and *S*_WH_ is the stripe component of WH.

In this study, the loss function of the network is

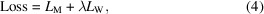

where λ is the regularization coefficient, which is used to balance the relationship between the two loss functions and prevent the network from under- or over-fitting.

## 3. Experiments and discussion

### 3.1. Evaluation of the RRAC method by synthetic data

The RRAC method is compiled in a Python environment, and the neural network is built on the PyTorch framework (Paszke *et al.*, 2019 ▸). All the tests are carried out on a workstation with a CPU of a 2.2 GHz Intel Xeon silver 4114 and a NVIDIA Quadro p6000 graphics processing unit.

The accuracy of the RRAC method is evaluated by synthetic data. Due to the lack of sufficient public CT datasets to train and test the network, the Div2k dataset is selected as the synthetic data, which is composed of many high-resolution 2D images (Agustsson & Timofte, 2017 ▸). The Div2k dataset is pre-processed as follows to establish training and test sets: first, 824 pre-processed slice images P_1_ are randomly picked up from the Div2k dataset, converted to grayscale images, and clipped. Second, images P_1_ (512 × 512) are Radon-transformed to obtain the ground truth sinogram image S_1_. (A total of 360 projective images were recorded over an angular range of 0° to 179.5°.) Finally, sinogram S_1_ is added with random stripe artifacts to acquire artifact sinogram image S_2_. In total, the training set includes 712 randomly selected groups of ground truth S_1_ and artifact sinogram images S_2_. The remaining 112 image groups are adopted as a test set to evaluate network accuracy. The training and test sets established as above not only solve the problem of insufficient data but also have better visibility of the artifact than CT data, which facilitate the subsequent evaluation. Moreover, compared with CT data, the better variety of the Div2k dataset brings the network more versatility. The degree of artifacts added manually is evaluated by the peak signal-to-noise ratio (PSNR). A high PSNR value means few artifacts in the image.

**Figure 3:**
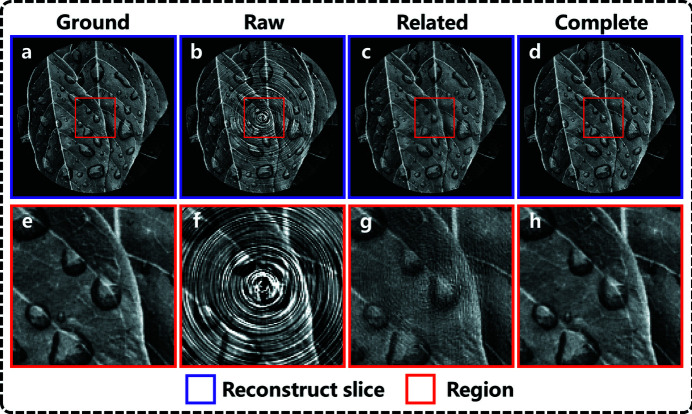
Reconstructed slice with ground truth (*a*), without correction (*b*), with related correction model (*c*), with complete correction model (*d*). (*e*–*h*) Magnified views of the selected regions.

The effect of complementary information of wavelet coefficients on artifact correction is evaluated through ablation experiments. The correction network is trained by approximation and horizontal wavelet coefficients, which is called the related artifact correction model, whereas training through all wavelet coefficients is called the complete artifact correction model. The correction results of these two models are shown in Fig. 3 ▸. By comparing reconstructed results it can be seen that, although the related correction model can remove the ring artifacts, it has a poor ability to restore and preserve the original details of the image. The result using the complete correction model is almost the same as the ground truth. The above results prove that the complete correction model can effectively preserve and restore detailed information while removing artifacts through complementary information of each wavelet coefficient.

**Figure 4:**
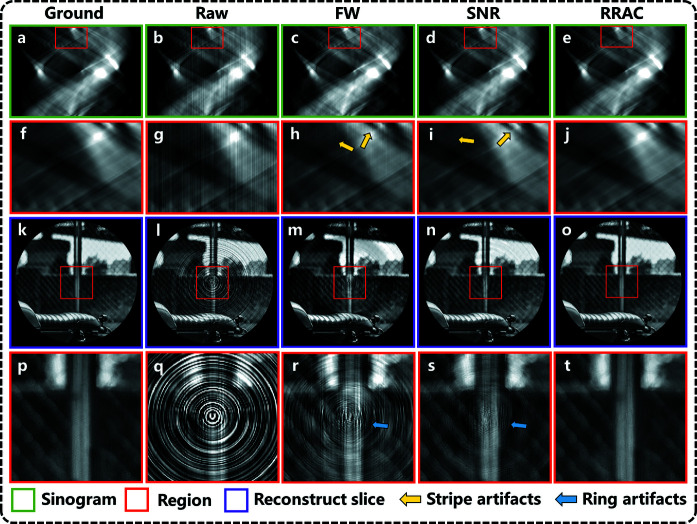
Correction results of different artifact removal methods for Div2k simulation data. Sinogram images (*a*) with ground truth, (*b*) without correction (PSNR: 13.51), (*c*) with the FW method, (*d*) with the SNR method, and (*e*) with the RRAC method. (*f*–*j*) Magnified sinogram images corresponding to the red squares shown in (*a*)–(*e*). (*k*–*o*) Sinogram images corresponding to slice images. (*p*–*t*) Magnified views of the selected regions shown in (*k*)–(*o*).

After training, the network can be applied to the test data to access its performance. The raw sinogram, as illustrated in Fig. 4 ▸(*b*), is obtained by adding the ground truth sinogram [Fig. 4 ▸(*a*)] with random stripe artifacts. Its stripe correction results using the FW, SNR and RRAC methods are displayed in Figs. 4 ▸(*c*), 4 ▸(*d*) and 3(*e*), respectively. Figs. 4 ▸(*f*)–4(*j*) show enlargements of the outlined areas in Figs. 4 ▸(*a*)–4(*e*). Compared with the unprocessed sinogram, an obvious reduction of stripes can be observed from sinograms processed by all three methods. However, the RRAC method has achieved the lowest level of residual stripes. Its sinogram is also fairly identical to that of the ground truth. The SNR method cannot remove some strong artifacts with a certain width, and the FW method is even more inferior. The corresponding slices reconstructed by the FBP algorithm are presented in Figs. 4 ▸(*k*)–4(*o*). Ring artifacts in slices are sensitive to the stripes in sinograms, even the invisible residual stripes after FW correction can produce strong rings in slices. The RRAC method can obtain the minimum and almost invisible ring artifacts and preserve the most image details of the slices.

**Figure 5:**
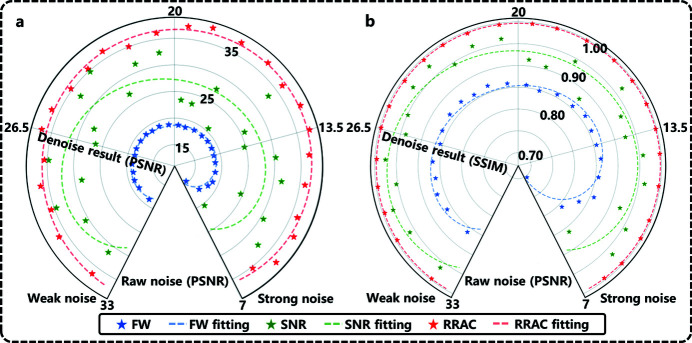
PSNR/SSIM results of various correction methods.

The evaluation is performed quantitatively to further explore the RRAC method. The PSNR and structural similarity coefficient (SSIM,0∼1) are chosen as evaluation criteria (Wang *et al.*, 2004 ▸). A high PSNR value means few artifacts in an image and high accuracy of the corresponding correction method, whereas a large SSIM value implies a good capability of preserving and restoring details during the correction process. The PSNR and SSIM values of different correction methods are calculated and shown in Fig. 5 ▸. Although the accuracy of the FW method for artifacts of different degrees is relatively stable, this method not only has low accuracy but also corrupts the details during the correction process. Although the SNR method shows better accuracy than the FW method, its accuracy is more vulnerable to artifact strength, so the robustness and versatility of the SNR method are inferior. Compared with previous methods, the RRAC method can obtain a more precise correction and more stable performance for different degrees of artifacts.

### 3.2. Application of the RRAC method by experimental data

The RRAC method has shown its advantages of high precision and performance regarding synthetic data. However, due to the significant difference between synthetic and experimental data, the network trained by simulated data is inapplicable to actual application directly. The network also cannot be well trained through limited experimental datasets. Thus, the transfer learning strategy has been adopted to solve experimental data insufficiency (Tan *et al.*, 2018 ▸; Zhang & Gao, 2019 ▸). This strategy is as follows: the network is initialized with the parameters trained by the large synthetic dataset and is then further trained with a small experimental dataset. This training strategy not only achieves excellent results under training data shortage but also greatly reduces the training difficulty. CT data of shale collected on the 4W1A station of Beijing Synchrotron Radiation Facility (Yuan *et al.*, 2012 ▸) are adopted to further explore the feasibility of the proposed method as a real-world application. This projection (512 × 512) is acquired from 361 angles over 180° at 8 keV by a micrometre-resolution X-ray microscope with an effective pixel size of 2.5 µm.

**Figure 6:**
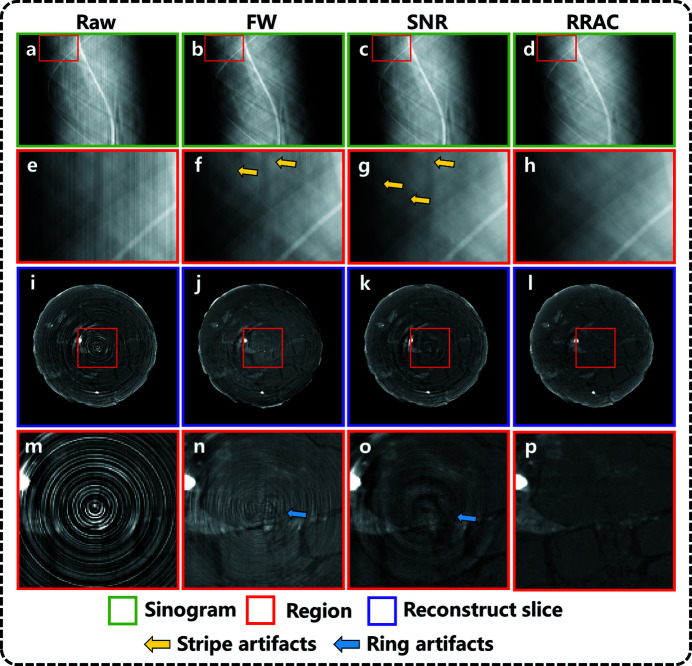
Correction results of different artifact removal methods for shale data. Sinogram images (*a*) without correction, (*b*) with FW correction, (*c*) with SNR correction, (*d*) with deep learning-based artifact correction. (*e*–*h*) Magnified sinogram images corresponding to the red squares shown in (*a*)–(*d*). (*i*–*l*) Sinogram images corresponding to slice images. (*m*–*p*) Magnified views of the selected regions shown in (*i*)–(*l*).

Fig. 6 ▸(*a*) shows the unprocessed raw sinogram image. Figs. 6 ▸(*b*), 6(*c*) and 6(*d*) are sinogram images corrected by the FW, SNR and proposed methods, respectively. Figs. 6 ▸(*e*)–6(*h*) show enlargements of the outlined areas in Figs. 6 ▸(*a*)–6(*d*). Reconstructed slice images [Figs. 6 ▸(*i*)–6(*l*)] are obtained from the FBP reconstruction. Figs. 6 ▸(*m*)–6(*p*) show enlargements of the outlined areas in Figs. 6 ▸(*i*)–6(*l*). Residual stripes can be found in the sinogram corrected by the FW method [Fig. 6 ▸(*b*)]. Subsequently, the image details of reconstructed slices [Fig. 6 ▸(*j*)] are corrupted by ring artifacts. In Fig. 6 ▸(*c*), most artifacts are removed using the SNR method, except some artifacts of a certain width, resulting in wide ring artifacts in some slices [Fig. 6 ▸(*k*)]. Referring to the RRAC method, almost no artifacts can be observed in the sinograms [Fig. 6 ▸(*d*)] and slices [Fig. 6 ▸(*l*)]. Furthermore, the RRAC method can preserve the fine structure of the shale when removing the stripes, so the resolution of the reconstructed slice images [Fig. 6 ▸(*l*)] is also greatly improved. In summary, the above experiment exhibits that after incorporating the transfer learning strategy the RRAC method also shows the remarkable capability of stripe artifact removal in the application of experimental data and outperforms the traditional method.

## 4. Conclusion

This study introduces a ring artifact correction method named RRAC, which is based on ResNet. Compared with the SNR method, ResNet is introduced, which is thought to be efficient in solving complex problems with deep networks. HDWT is also incorporated to decompose the sinogram into complementary coefficients before being fed into the network. In the synthetic data experiment, the combination of residual network and HDWT exhibits better capability of removing ring artifacts than the reference methods while fully preserving image details. Given that a large training dataset comprising experimental data is unavailable, the transfer learning strategy enables network training with limited experimental data. To reasonably evaluate its accuracy and performance, the RRAC method is evaluated by synthetic and experimental data. When applied to synthetic data, the RRAC method prevails on accuracy and detail preservation in visual and quantitative comparisons with other methods. Moreover, through the experiment on real CT data, the transfer learning strategy succeeds in maintaining the superiority of the RRAC method over the other methods without the availability of abundant experimental data for training. In summary, our proposed method is effective and adaptable to various data types with minimal training data requirements. Its application helps further improve CT 3D reconstruction quality and facilitate subsequent data analysis.
